# Spatial-temporal clustering of an outbreak of SARS-CoV-2 Delta VOC in Guangzhou, China in 2021

**DOI:** 10.3389/fpubh.2022.1050096

**Published:** 2022-12-09

**Authors:** Qian Zhang, Meng Zhang, Jianxiong Hu, Guanhao He, Yan Zhou, Xuguang Chen, Yali Zhuang, Zuhua Rong, Lihua Yin, Jianguo Zhao, Zitong Huang, Weilin Zeng, Xing Li, Zhihua Zhu, Yerong Tang, Yi Quan, Yihan Li, Li Zhang, Di Fu, Yan Li, Jianpeng Xiao

**Affiliations:** ^1^School of Public Health, Guangdong Pharmaceutical University, Guangzhou, China; ^2^Guangdong Provincial Institute of Public Health, Guangdong Provincial Center for Disease Control and Prevention, Guangzhou, China; ^3^Guangdong Provincial Center for Disease Control and Prevention, Guangzhou, China; ^4^Guangdong Workstation for Emerging Infectious Disease Control and Prevention, Guangzhou, China; ^5^School of Public Health, Southern Medical University, Guangzhou, China; ^6^School of Public Health, Sun Yat-sen University, Guangzhou, China; ^7^Arboviral Disease Prevention Department, Yunnan Institute of Parasitic Diseases, Puer, China; ^8^Department of Public Health and Preventive Medicine, School of Medicine, Jinan University, Guangzhou, China

**Keywords:** SARS-CoV-2 Delta, Knox analysis, spatial-temporal clustering, intervention measures, COVID-19

## Abstract

**Background:**

In May 2021, the SARS-CoV-2 Delta variant led to the first local outbreak in China in Guangzhou City. We explored the epidemiological characteristics and spatial-temporal clustering of this outbreak.

**Methods:**

Based on the 153 cases in the SARS-CoV-2 Delta variant outbreak, the Knox test was used to analyze the spatial-temporal clustering of the outbreak. We further explored the spatial-temporal clustering by gender and age groups, as well as compared the changes of clustering strength (S) value between the two outbreaks in Guangzhou.

**Results:**

The result of the Knox analysis showed that the areas at short distances and brief periods presented a relatively high risk. The strength of clustering of male-male pairs was higher. Age groups showed that clustering was concentrated in cases aged ≤ 18 years matched to 18–59 years and cases aged 60+ years. The strength of clustering of the outbreak declined after the implementation of public health measures. The change of strength of clustering at time intervals of 1–5 days decreased greater in 2021 (S = 129.19, change rate 38.87%) than that in 2020 (S = 83.81, change rate 30.02%).

**Conclusions:**

The outbreak of SARS-CoV-2 Delta VOC in Guangzhou has obvious spatial-temporal clustering. The timely intervention measures are essential role to contain this outbreak of high transmission.

## Introduction

Coronavirus disease 2019 (COVID-19), associated with severe acute respiratory syndrome coronavirus-2 (SARS-CoV-2) infection outbroke in December 2019. Since the outbreak of the pandemic, several prevalent SARS-CoV-2 mutant strains have been encountered globally, resulting in a considerable socioeconomic burden (1). As of October 2022, the World Health Organization (WHO) has identified five main lineages as variables of concern (VOC), including Alpha, Beta, Gamma, Delta, and Omicron (2, 3). The emergence of SARS-CoV-2 variants has accelerated the spread of COVID-19 (4). For instance, the Delta VOC (B.1.617.2) had rapidly outcompeted other variants of SARS-CoV-2 within several months and became a dominant variant in the third wave of the COVID-19 pandemic (5–7). With the continuous mutation of the virus, the transmission capacity may be enhanced, bringing many uncertainties to the future prevention and control of the epidemic. Learning the lesson from the transmission characteristics of the Delta VOC will administer to the response to the next COVID-19 outbreak.

The transmission of COVID-19 has obvious characteristics of spatial-temporal clustering. Some studies have found that appropriate public intervention measures can be effective in reducing the transmission of COVID-19 (8–10). Several studies have explored the spatial-temporal characteristics of COVID-19 by combining the time and space scale (1, 11), while few studies have considered the intervention measures at different stages, such as before and after the peak of the outbreak. In addition, scholars generally investigated the transmission characteristics of COVID-19 at the national or city scale, but few studies investigated it at a small space level (such as street or community level), which would provide limited information for the refined measures making. Several studies have shown that the contact pattern between different populations also plays an important role in the transmission of COVID-19. Thus, studying the spatial-temporal clusters of COVID-19 by considering contact patterns between the different populations at the street level and by the stages of intervention measures, would help us to identify risk populations and assess the effect of intervention measures.

China maintains a strict policy of public health interventions in response to the COVID-19 outbreak. On 21 May 2021, the first local outbreak in China from the highly transmissible Delta variant was identified in Guangzhou, Guangdong Province (12). Guangzhou implemented a series of strict public health measures, including lockdown of high-risk areas, isolation of contacts, and mass nucleic acid screening. Despite the rapid spread of the Delta variant, the outbreak was contained within a month (11). In addition, the cases from this outbreak have a very clear transmission chain. The outbreak and response of the Delta VOC in Guangzhou offer a unique opportunity for a better understanding of the high transmissible and adaptation measures of the Delta VOC. In this study, the Knox test was used to explore the spatial-temporal cluster characteristics, identify the high-risk groups, and discover the changes in spatial-temporal clustering with different intervention stages. The findings would provide a reliable reference for effectively responding to the next SARS-CoV-2 variant outbreak.

## Materials and methods

### Data collection

#### COVID-19 cases

Data on COVID-19 cases reported in Guangzhou from April 2 to May 4, 2020, and from May 18 to June 18, 2021, were obtained from the Guangdong Center for Disease Control and Prevention (Guangdong CDC). COVID-19 cases were individuals with a positive result in a PCR for SARS-CoV-2 in respiratory specimens, referring to guidelines issued by Chinese National Health Commission (13) and WHO (14). Information for each case included gender, age, address, diagnosis, date of illness onset, and type of case (imported case or local case).

#### Intervention measures

Data and timelines for relevant public health interventions for the Delta VOC outbreak were obtained from the emergency response working group of Guangdong CDC and the website of Guangzhou Municipal Health Commission (http://wjw.gz.gov.cn/).

#### Map and population data

The map data of the administrative interface of each street in Guangzhou were obtained from the Data Center of Resource and Environmental Science (http://www.resdc.cn/), and demographic information of each street in Guangzhou was obtained from the Statistical Yearbook of Guangdong Province (http://stats.gd.gov.cn).

### Knox test

Spatial-temporal interaction refers to the phenomenon that events located relatively close in space occur also close in time (15). The Knox test (16) is a method that is used frequently to detect this kind of interaction. It has been used in many areas, such as criminology (17), medical and public health (18), and epidemiology (19–22). Its basic principle and form are as follows: the null hypothesis of the Knox method is that the occurrence of diseases is spatial-temporal clustering. All possible pairs of cases are assembled and classified according to their distances apart in space and time (21). The observed number of pairs within short space and short time intervals is compared with the number expected if the space and time intervals between pairs are independent of each other (23). The test statistic, *X*, is the number of pairs of cases that are close in both space and time. The statistic is calculated as:


(1) $$\text{X}(s,t)=\sum_{i=1}^{N}\sum_{j=\;1}^{N}a_{ij}^{s}a_{ij}^{t}$$

where *N* is the number of cases, $a_{ij}^{s}$ is equal to 1 if cases *i* and *j* are close in space, and 0 otherwise, $a_{ij}^{t}$ is equal to 1 if cases *i* and *j* are close in time and 0 otherwise, and *s* and *t* represent pre-specified spatial and temporal distances (21, 24).

The most commonly used method, Monte Carlo hypothesis testing, was proposed by Mantel (25). If the difference between statistic *X* and its expected value is statistically significant, it is considered to be spatial-temporal clustering (15). For known infectious diseases, time and space boundaries can be selected by referring to the incubation period and transmission characteristics. Based on our knowledge of the COVID-19 transmission pattern and previous cluster analyses of COVID-19 transmission, the Knox test is used to examine spatial-temporal interactions from 0 meters to 1,000 kilometers at time intervals from 1 to 14 days (16, 20, 23). In this study, 1 day was selected as the time interval referenced from previous reports (26). The space distance is divided into 10 intervals, the interval is 100 meters. And the relative risk (RR) was used to represent the risk of COVID-19 infection under this spatial-temporal threshold value compared with other conditions. We used *S* to represent the strength of spatial-temporal clustering under the conditions of various spatial-temporal critical values. The sum of statistically significant *S* values (Σ*S*) under various spatial-temporal interval combinations was set as the total strength of spatial-temporal clustering.

In the Knox analysis, we Geo-coded for each case in the Delta outbreak first. We converted the residential address of each infection case into latitude and longitude coordinates by the Baidu map coordinate picker (http://api.map.baidu.com/lbsapi/getpoint/index.html), subsequently, the projection coordinate system of all the coordinates were converted to WGS84 projection Coordinate System (World Geodetic System 1984).

In this study, we first investigated the spatial-temporal clustering of the overall cases during the whole epidemic period, then we compared the clustering for different case pairs which represented different contact patterns. All case pairs were divided by gender into the male match to male (male-male), female-female, and male-female. All case pairs were divided into six groups based on age, including aged ≤ 18 years, aged 18–59 years, aged 60+ years; and aged ≤ 18 years–aged 18–59 years, aged ≤ 18 years–aged 60+ years, and aged 18–59 years–aged 60+ years.

To further address the effectiveness of public health interventions in the Delta outbreak, we divided the timing of this outbreak into two the stages. The first stage is from 18 May to 31 May 2021, and the second stage is from 1 June to 18 June 2021. Most public health interventions were implemented in the first phase. In addition, we included another COVID-19 epidemic which has a similar scale to the epidemic in Guangzhou as the comparative case. We included 215 cases of local outbreaks in Guangzhou from April to May 2020 (27), and compared the changes in strength (S) of spatial-temporal clustering between the two outbreaks.

All data were analyzed by R software, version 4.1.0 (http://CRAN.R-project.org, R Foundation, Vienna, Austria), and the “surveillance” package (28) was used to conduct the Knox test. *P*-value < 0.05 was considered statistically significant.

## Results

### Spatial-temporal distribution

**Figure 1:**
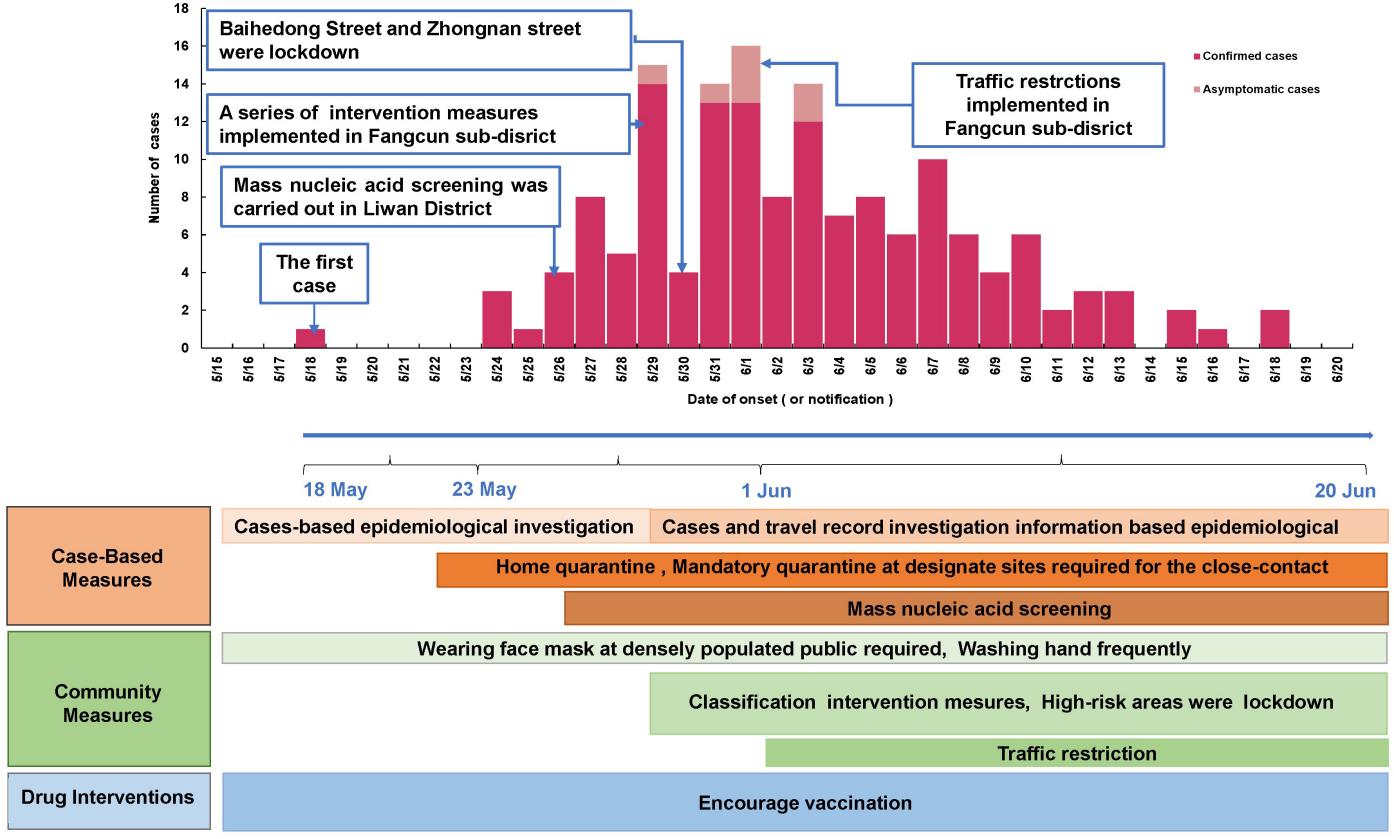
Epidemic curve of and the intervention measures against SARS-CoV-2 Delta VOC outbreak in Guangzhou, from May 18, 2021 to June 18, 2021.

As of 19 June 2021, a cumulative total of 153 COVID-19 cases were reported in Guangzhou (Figure 1). The first case was diagnosed on May 21, 2021, and the number of cases has increased markedly since May 26, 2021. The peak daily number of cases was 16 on June 1, 2021, then, the incidence subsequently decreased since June 1, 2021, and no more related cases were reported from June 19, 2021.

**Figure 2:**
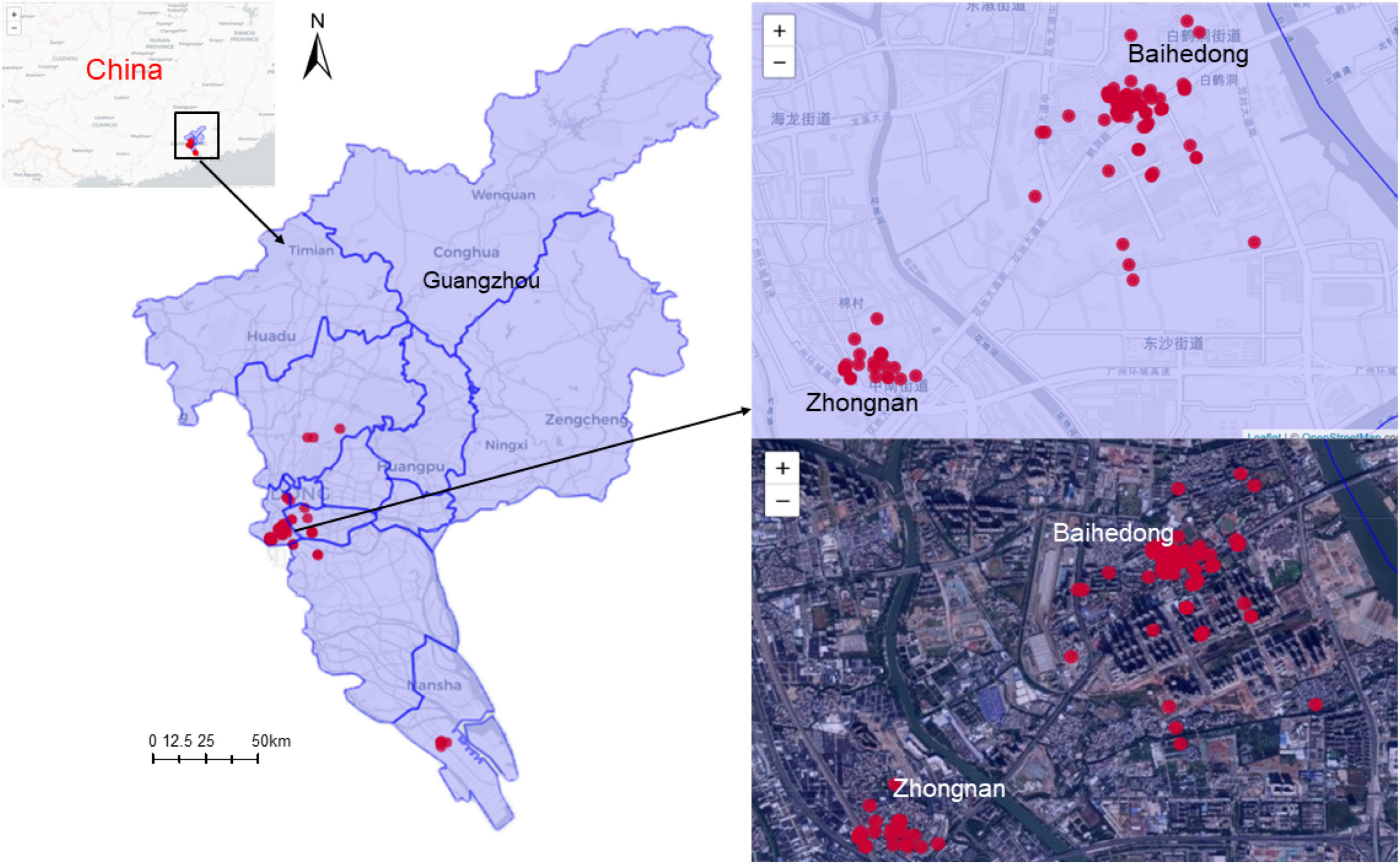
Spatial distribution of SARS-CoV-2 Delta VOC outbreak in Guangzhou, from May 21, 2021 to June 18, 2021.

Figure 2 shows the distribution of the cases in Guangzhou. The cases were distributed in 17 streets/towns in six districts, mainly concentrated in Baihedong Street and Zhongnan Street in Liwan District (89 cases), while Chongkou street and Dongsha Street had only one case each. Overall, the spatial distribution of confirmed cases varies significantly.

The epidemic curve and the response process of this outbreak are shown in Figure 1. Since the first case was detected on May 21, 2021, Guangzhou took a series of intervention measures immediately (29), including epidemiological investigation, quarantine at designated sites required for close contact, precise and differentiated epidemic prevention and control strategies, mass nucleic acid screening, and encouragement of vaccination (Figure 1). On May 22, the community where the first confirmed case lived was designated as a medium-risk area, and nucleic acid screening for key populations began on May 26. On May 29, 2021, strictly implement intervention measures at different levels and risk areas. Areas such as Baihedong Street and Zhongnan Street were on lockdown. In addition, traffic restrictions enhanced in Guangzhou since June 1, 2021.

### The spatial-temporal clustering of all case pairs (0–1,000 m, 1–14 d)

**Figure 3:**
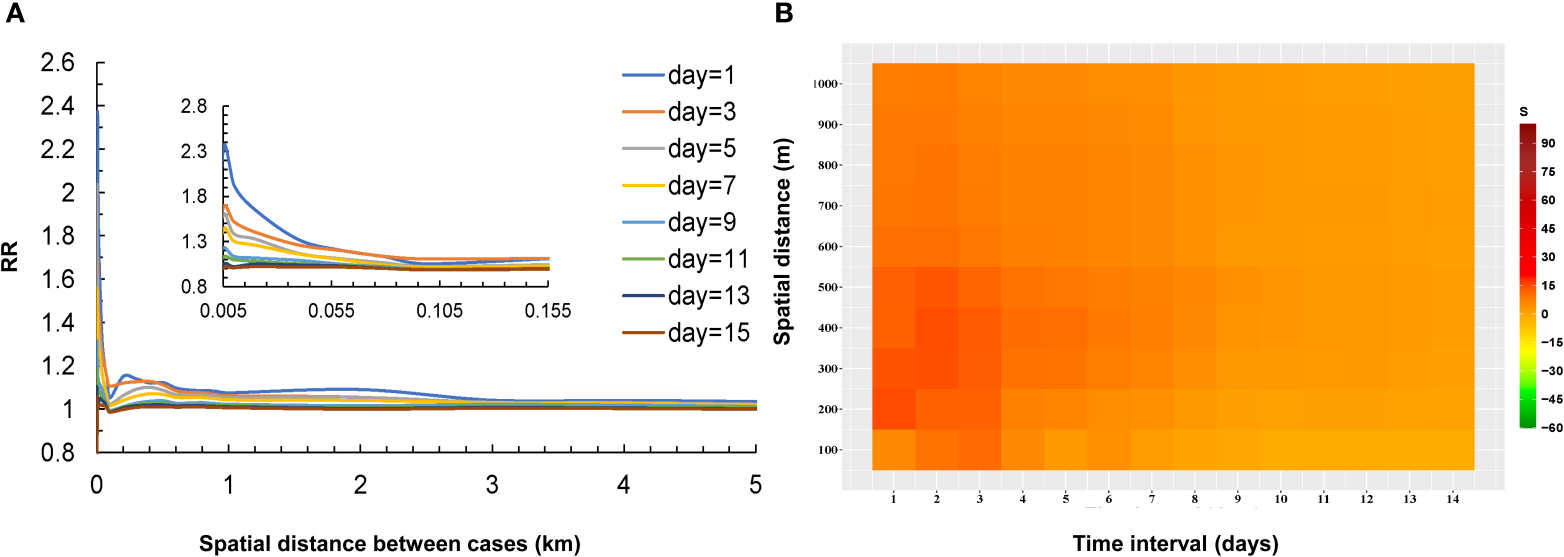
Relationship between the risk of infection and distance for COVID-19 case pairs at different temporal thresholds in Guangzhou **(A)**, the strength of spatial-temporal clustering of all case pairs (≤1,000 m and ≤14 d) **(B)**.

The result of the Knox test analysis showed a significant spatial-temporal interaction within the time interval of 1–14 days and the spatial distance of 0–1,000 m. The average time interval of all case pairs was 6.2 days, and the average spatial distance was 7.6 km. The RR declined with the distance increased between the two cases and the RR decreased gradually as the time between the onset of the two cases prolonged. The RR reached 2.3 at very short distances at time intervals of 1 day. When the distance increased to 1 km, the RR rapidly declined to 1.1 and approached 1 at a distance of 5 km. The risk of infection at other time intervals also showed a similar pattern in Figure 3A.

The *S* values (the strength of the spatial-temporal clustering) are shown in Table 1. According to the Monte Carlo test, most *S* values were statistically significant (*P* < 0.05). The highest levels of strength of the spatial-temporal clustering occurred at intervals of 0 and 1 day and within 100 meters (Figure 3B). The areas at short distances within 550 meters and brief periods within 5 days presented high strength of the spatial-temporal clustering (*S* > 15, most of the S values were >30). With the distance between the two cases increased, the *S* values decreased. The *S* values also gradually decreased as the onset interval between the two cases was prolonged.

**Table 1:** The strength of spatial-temporal clustering (S) of all case pairs from May to June 2021 in Guangzhou, China.

**Time interval (days)**	**Distance between case pairs (meters)**									
	**100**	**200**	**300**	**400**	**500**	**600**	**700**	**800**	**900**	**1000**
1	**116.4**	**82.16**	**81.33**	**67.93**	**55.07**	**44.27**	**33.52**	**31.28**	**28.01**	**28.41**
2	**96.26**	**72.64**	**67.61**	**52.39**	**39.94**	**33.12**	**25.53**	**22.37**	**20.88**	**19.22**
3	**64.78**	**47.84**	**45.30**	**30.64**	**25.53**	**18.76**	**14.12**	**14.19**	**12.10**	**9.63**
4	**53.19**	**43.23**	**39.00**	**28.63**	**22.13**	**17.60**	**13.69**	**12.82**	**9.99**	**8.16**
5	**39.99**	**29.67**	**25.90**	**19.65**	**15.66**	**11.90**	**7.89**	**8.74**	**6.99**	**6.96**
6	**30.55**	**21.46**	**18.04**	**12.17**	**9.96**	**7.11**	3.45	4.70	3.64	4.11
7	**25.43**	**15.99**	**13.79**	**8.06**	**6.17**	3.53	1.18	1.30	0.05	0.73
8	**21.03**	**12.52**	**10.08**	**4.70**	3.55	1.17	−0.77	−0.95	−1.60	−1.07
9	**17.92**	**11.67**	**8.53**	**4.99**	3.66	0.88	−0.99	−1.24	−1.73	−1.16
10	**15.10**	**9.72**	**6.95**	4.28	3.31	1.23	−0.36	−0.68	−1.04	−0.42
11	**13.57**	**7.63**	**4.94**	2.96	2.23	0.73	−0.92	−1.18	−1.49	−0.98
12	**10.41**	**5.19**	2.71	0.70	0.19	−1.35	−2.15	−2.12	−2.61	−1.56
13	**6.84**	2.47	0.91	−0.32	−0.97	−1.84	−1.58	−1.56	−1.86	−0.95
14	**5.07**	2.37	0.81	−0.92	−1.23	−1.89	−1.50	−1.43	−1.50	−0.63

The number in bold font indicates that Strength (*S*) is were statistically significant (*P* < 0.05).

### Spatial-temporal clustering of cases with different characteristics

**Figure 4:**
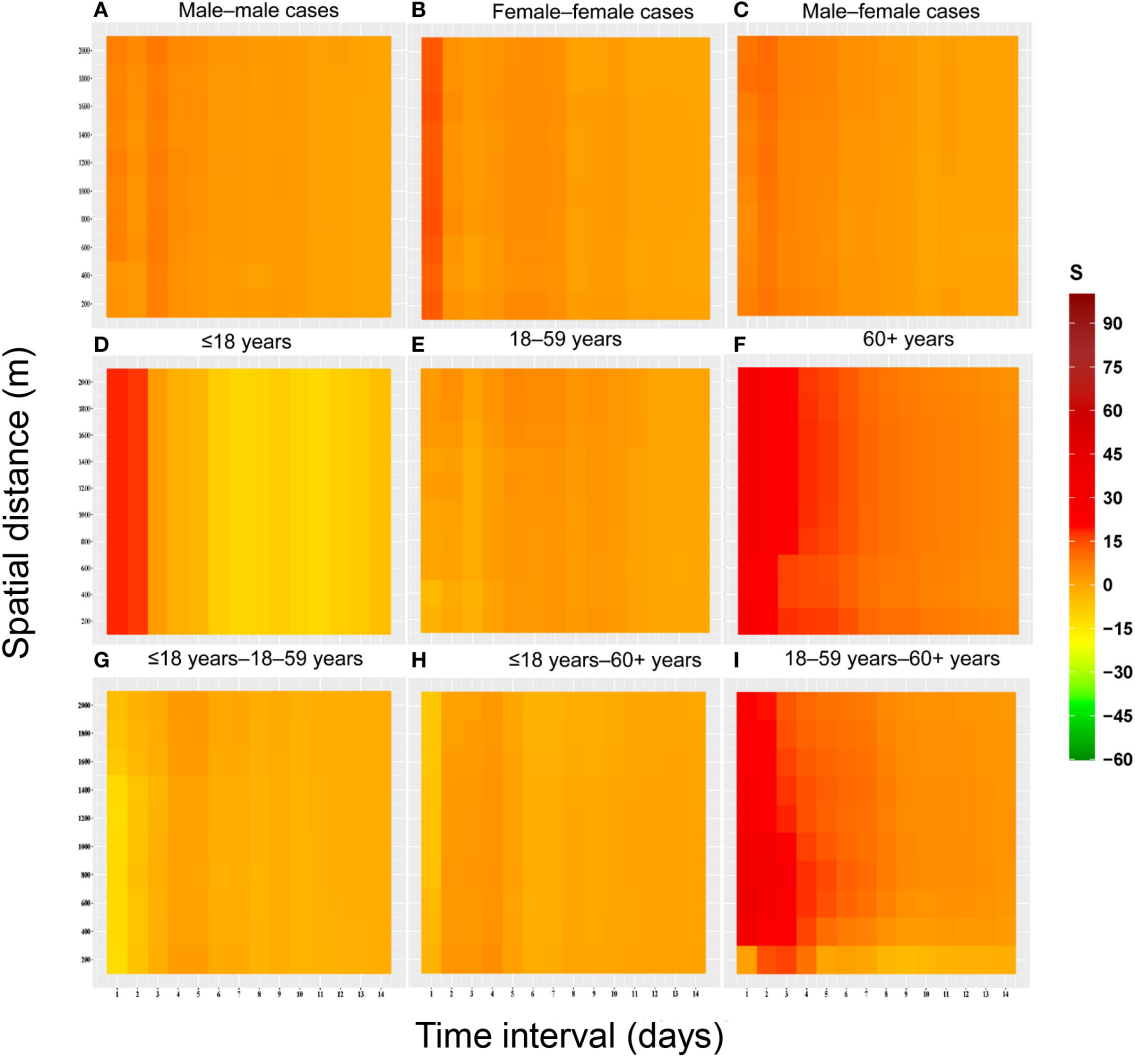
Heat map of spatial-temporal clustering due to space-time interactions among populations with different characteristics for the COVID-19 epidemic in Guangzhou from May to June 2021. **(A)** Male–male cases. **(B)** Female–female cases. **(C)** Male–female cases. **(D)** ≤18 years. **(E)** 18–59 years. **(F)** 60+ years. **(G)** ≤18 years−18–59 years. **(H)** ≤18 years−60+ years. **(I)** 18–59 years−60+ years.

The results showed that spatial-temporal clustering of the COVID-19 cases was similar between the genders at the 0–1,000 m scale, and all showed a trend of decreasing the strength of spatial-temporal clustering (*S*) with increasing time thresholds and spatial distance. The strength of spatial-temporal clustering of male-male pairs was higher at time thresholds of 1–7 days and spatial distances < 600 m. The strength of the spatial-temporal clustering showed that male-male > male-female > female-female (Figure 4).

According to the results, the clustering was greater in case pairs aged ≤ 18 years–aged 18–59 years, and cases pairs aged 60+ years at short time intervals (1–5 days). Three peaks of clustering were observed for the case pairs aged ≤ 18 years–aged 18–59 years, at 1, 3, and 6 days, respectively. In addition, the pattern of spatial-temporal clustering characteristics in the case pairs aged 18–59 years was similar to that in the genders.

**Figure 5:**
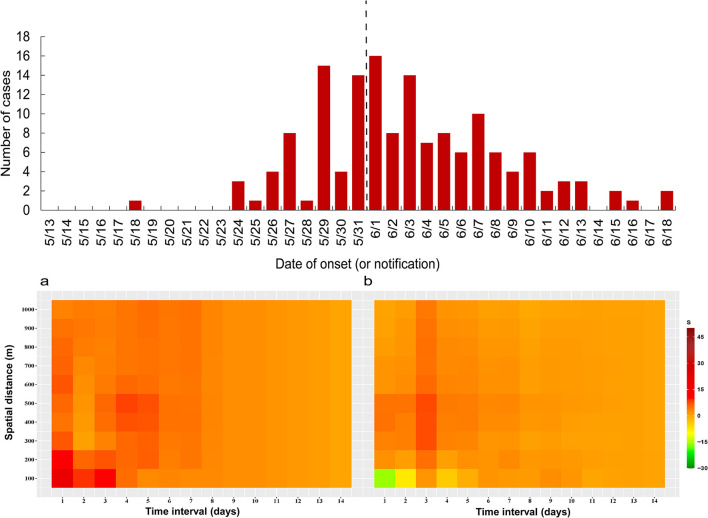
Spatial-temporal clustering of all cases before and after taking interventions (1,000 m), **(a)** May 18 to May 31, 2021, **(b)** June 1 to June 18, 2021.

The difference in spatial-temporal clustering of all cases before and after the peak of the epidemic is presented in Figure 5. The strength of spatial-temporal clustering before the peak of the epidemic was greater than that after the peak of the epidemic. Before the peak of the epidemic (May 18 to May 31, 2021), the maximum *S* value was 26, and the sum of *S* values was 541, and after the peak of the epidemic (June 1 to June 18, 2021), the maximum *S* value was 9, and the sum of *S* values was 304 (Supplementary Table S1). Before the peak of the outbreak, the spatial-temporal clustering within 300 m is the strongest, especially on the first and third days, the spatial-temporal cluster within 100 m is higher than that under other spatial-temporal boundaries. In particular, the spatial-temporal clustering at 1 and 3 days in the 100 m range were higher than other spatial-temporal scales.

**Figure 6:**
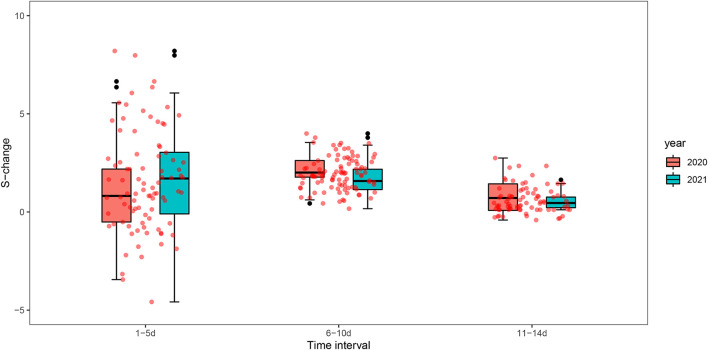
Box plots of spatial-temporal clustering changes (*S*-change) before and after the peak of the epidemic in Guangzhou in 2020 and 2021.

Figure 6 and Supplementary Table S2 show the changes in *S* value before and after the peak between the two outbreaks in Guangzhou in 2020 and 2021, we found that the change in the strength of spatial-temporal clustering decreased greater in 2021 (*S*-change = 129.19, change rate of 38.87%) than in 2020 (*S*-change = 83.81, change rate of 30.02%) at time intervals of 1–5 days, while at 6–10 days and 11–14 days, the changes in *S* value were greater in 2020.

## Discussion

In this study, we characterized the spatial-temporal clustering of the COVID-19 outbreak in Guangzhou from May 21, 2021, to June 18, 2021, based on detailed individual information on each case. This outbreak is the first community-transmitted outbreak of the Delta VOC in China. Our study found that the outbreak of the Delta variant in Guangzhou has obvious spatial-temporal clustering, and effective interventions can reduce the spatial-temporal clustering, which may provide references for the response to the COVID-19 outbreak. We observed that the effect of spatial-temporal interaction was obvious and exhibited interval heterogeneity. The areas with the highest level of elevated risk were all within a distance of 1 kilometer. The highest risk value occurred at intervals of 0 and 1 day and gradually decreased as the onset interval was prolonged. This result indicated that COVID-19 infection from one case to the other individuals tends to occur in nearby persons. On the one hand, the Delta VOC B.1.617.2 grew faster and was more transmissible. Campbell et al. reported a 97% increase in the effective reproductive number of B.1.617.2 by 3 June 2021 (30). This may be due to the following reasons: (1) Guangzhou has a dense population, convenient transportation, and frequent personnel exchanges, which have naturally facilitated the spread of the virus (30). (2) In the early stage of the epidemic, five generations of transmission occurred within 10 days, and the effective reproduction number (Rt) increased to 7.1 on May 27, 2021 (28), resulting in a rapid increase in the number of cases in the early stage, with strong spatial-temporal clustering. On the other hand, the virus could transmit through short-distance contacts, such as meals, family contact, and community contact (1). It suggested that daily protection, such as wearing masks and getting vaccinated, should be taken in case of the high spreading risk of the COVID-19 Delta VOC.

We also found that the spatial-temporal clustering of different populations is different. The clustering strength of male-male pairs was high at time intervals of 1–7 days and spatial distances < 600 m. The strength of spatial-temporal clustering (*S*-value) for case pairs showed a male-male > male-female > female-female pattern. It may be related to the higher infection rate for males (31, 32), the male population has more social interactions, and the awareness of self-protection is weaker. In terms of age, all age groups are infected, indicating that people of all ages are generally susceptible. The spatial-temporal clustering of case pairs aged ≤ 18 years–aged 18–59 years and cases pairs aged 60+ years were higher, and the clustering strengths of infection among adolescents and the elderly were higher. Moreover, the spatial-temporal clustering of adolescents was strong in a short time interval (1–5 days). In the outbreak, the transmission sites of the COVID-19 Delta virus are mainly confined spaces, with obvious family clustering (29). Intra-family transmission may be related to the increase in cases since the restrictions of human movement (33–35). Thus, the pairs of patients aged 18–59 and over 60 years were strongly clustered in the family. In June 2021, the Delta VOC was also reported to be the main circulating strain in the United Kingdom, with the transmission in primary and secondary schools (36). All of the critically ill cases were in patients aged over 60 years, indicating that older patients are more susceptible to Delta VOC infection (37). In the face of the continuous mutation of the virus, it is necessary to strengthen the protection of young people and the elderly.

This study shows the difference in spatial-temporal clustering of all cases before and after the peak of the epidemic. The spatial-temporal clustering before the epidemic peak is stronger than that after the peak. Scientific prevention and control measures are the keys to preventing the spread of the epidemic. Due to the implementation of a series of intervention measures in Guangzhou in the early stage, the spatial-temporal clustering of all cases dropped significantly after June 1, 2021. In response to the significantly greater transmissibility in community settings, strict measures are taken against the spread of Delta VOC during the outbreak in Guangzhou. First, tens of millions of massive citywide viral RNA screening tests have been launched, targeting both the general and high-risk populations. Second, key risk areas were lockdown in time. Third, a detailed epidemiology investigation and a big data-based QR code system helped identify close contacts of cases. With these efforts, the outbreak was quickly contained within 1 month. These experiences could be valuable for the containment of emerging widespread variants.

We found that the strength of spatial-temporal clustering in time intervals (1–5 days) has decreased greater in 2021 than that in 2020. The reason may be that more stringent interventions were adopted in the early stage of the outbreak in 2021. It was reported that the incubation period of Delta is shorter (about 4 days) (38, 39), which needs to be an urgent response. Guangzhou had taken a series of rigorous intervention measures timely including mass testing, active case finding, travel restrictions, and area lockdown to contain this outbreak. The finding further verifies that the public health intervention response to the Delta outbreak is timely and effective. This suggests that the implementation of rapid and rigorous interventions is the key to containing the Delta epidemic of high transmission.

Our study is based on the coordinate information of each case, which can accurately address the spatial-temporal clustering of a COVID-19 outbreak at a small scale and it may serve as a template for responding to small-scale outbreaks. Several limitations should be noted within our study. First, in the spatial-temporal analysis, the distance between the cases was computed by their usual living address. The actual site where the case contracted the virus may be a working location or a public place, such as mass transit and restaurants. The risk areas of the distance interval may be biased. Second, the study sample size which includes 153 cases is not very great, while all the cases had very clear transmission chains based on the detailed epidemiology survey. Third, about 20% of the population was fully vaccinated, and the vaccine may influenced the spread of the virus to some extent.

## Conclusion

Our study revealed differences in spatial-temporal clustering in different stages of the epidemic and among populations with different characteristics. Adolescents and seniors are the key groups we need to focus on. The results give strong evidence that timely public health interventions including rapid tracing, quarantine, keeping social distance, and nucleic acid screening are crucial to contain the outbreak of emergent high transmissible variants. Overall, our study provides a template for the clustering analysis of the ongoing COVID-19 epidemic and is informative for response to the next SARS-CoV-2 variant outbreak.

## Data availability statement

The raw data supporting the conclusions of this article will be made available by the authors, without undue reservation.

## Author contributions

QZ and JX wrote the manuscript. YL and JX conceived and designed the study and reviewed and revised the manuscript. XC, YLZ, MZ, QZ, and JX contributed to data collection and statistical analysis. JH, GH, YZ, ZR, LY, JZ, YQ, ZH,WZ, XL, ZZ, YT, YHL, LZ, and DF contributed to data visualization. All authors contributed to the article and approved the submitted version.

## References

[1] Wang Y Chen R Hu F Lan Y Yang Z Zhan C. Transmission, viral kinetics and clinical characteristics of the emergent SARS-CoV-2 Delta VOC in Guangzhou, China. EClinicalMedicine. (2021) 40:101129. 10.1016/j.eclinm.2021.101129 34541481 PMC8435265

[2] World Health Organization (WHO). Update on SARS-CoV-2 Variant Nomenclature. Available online at: https://www.who.int/publications/m/item/update-60-sars-cov-2-nomenclature-variants (accessed May 12, 2022).

[3] World Health Organization (WHO). Technical Advisory Group on Virus Evolution. Update on Omicron. Available online at: https://www.who.int/news/item/28-11-2021-update-on-omicron (accessed May 15, 2022).

[4] Hamamoto Y. The COVID-19 world - Are we there yet? J Diabetes Investig. (2021) 12:1125–7. 10.1111/jdi.13605 34056843 PMC8242779

[5] World Health Organization (WHO). Weekly epidemiological update on COVID-19. Geneva: WHO (2021). Available online at: https://www.who.int/publications/m/item/weekly-epidemiological-update-on-covid-19-−9-november-2021 (accessed June 1, 2022).

[6] Pascarella S Ciccozzi M Zella D Bianchi M Benedetti F Benvenuto D. SARS-CoV-2 B1617 Indian variants: are electrostatic potential changes responsible for a higher transmission rate? J Med Virol. (2021) 93:6551–6. 10.1002/jmv.27210 34260088 PMC8426736

[7] *Delta Strain Becomes Dominant Variant in U.S*. Available online at: http://en.people.cn/n3/2021/0708/c90000-9869504.html (accessed July 8, 202).

[8] Lai S Ruktanonchai NW Zhou L Prosper O Luo W Floyd JR. Effect of non-pharmaceutical interventions to contain COVID-19 in China. Nature. (2020) 585:410–3. 10.1038/s41586-020-2293-x 32365354 PMC7116778

[9] Liu K Ai S Song S Zhu G Tian F Li H. Population movement, city closure in Wuhan, and geographical expansion of the COVID-19 infection in China in January 2020. Clin Infect Dis. (2020) 71:2045–51. 10.1093/cid/ciaa422 32302377 PMC7188136

[10] Kraemer MUG Yang CH Gutierrez B Wu CH Klein B Pigott DM. The effect of human mobility and control measures on the COVID-19 epidemic in China. Science. (2020) 368:493–7. 10.1126/science.abb4218 32213647 PMC7146642

[11] Kang M Xin H Yuan J Ali ST Liang Z Zhang J. Transmission dynamics and epidemiological characteristics of SARS-CoV-2 Delta variant infections in Guangdong, China, May to June 2021. Euro Surveill. (2022) 27:1–10. 10.2807/1560-7917.ES.2022.27.10.2100815 35272744 PMC8915401

[12] Guangzhou Municipal Health Commission. COVID-19 Situation Update in Guangzhou (2021). Available online at: http://wjw.gz.gov.cn/ztzl/xxfyyqfk/yqtb/content/post_7295197.html (accessed July 28, 2021).

[13] National Health Commission & State Administration of Traditional Chinese Medicine. Diagnosis and Treatment Protocol for Novel Coronavirus Pneumonia (Trial Version 8) (2020). Available online at: http://www.nhc.gov.cn/ (accessed December 22, 2021). 10.1097/IM9.0000000000000022 PMC7227203 40479241

[14] World Health Organization. COVID-19 Clinical Management: Living Guidance (2021). Available online at: https://www.who.int/publications/i/item/WHO-2019-nCoV-clinical-2021-1 (accessed May 16, 2022).

[15] Jacquez GM. A k nearest neighbour test for space-time interaction. Stat Med. (1996) 15:1935–49. 10.1002/(SICI)1097-0258(19960930)15:18<1935::AID-SIM406>3.0.CO;2-I 8888486

[16] Knox EG Bartlett MS. The detection of space-time interactions. Series C Appl Stat. (1964) 1964:25–30. 10.2307/2985220

[17] Kalantari M Yaghmaei B Ghezelbash S. spatial-temporal analysis of crime by developing a method to detect critical distances for the Knox test. Int J Geogr Inf Sci. (2016) 30:2302–20. 10.1080/13658816.2016.1174867

[18] Waller LA Gotway CA. Applied Spatial Statistics for Public Health Data. Shanghai: Wiley-Interscience (2004).

[19] Lawson A. Statistical Methods in Spatial Epidemiology. New York, NY: John Wilcy & Sons (2013).

[20] Liu Q Li X Feng Z. Study on the appllcation of Knox method to temporal-spatial cluster for infectious disease. Chin J Epidemiol. (2007) 28:802–5. 18080571

[21] Jared A. An incremental Knox test for the determination of the serial interval between successive cases of an infectious disease. Stochast Environ Res Risk Assess. (2007) 21:487–500. 10.1007/s00477-007-0132-3

[22] Tran A Deparis X Dussart P Morvan J Rabarison P Remy F. Dengue spatial and temporal patterns, French Guiana, 2001. Emerg Infect Dis. (2004) 10:615–21. 10.3201/eid1004.030186 15200850 PMC3323097

[23] Gilman EA McNally RJ Cartwright RA. Space-time clustering of acute lymphoblastic leukaemia in parts of the U.K. (1984-1993). Eur J Cancer. (1999) 35:91–6. 10.1016/S0959-8049(98)00345-1 10211094

[24] Aldstadt J Yoon IK Tannitisupawong D Jarman RG Thomas SJ Gibbons RV. Space-time analysis of hospitalised dengue patients in rural Thailand reveals important temporal intervals in the pattern of dengue virus transmission. Trop Med Int Health. (2012) 17:1076–85. 10.1111/j.1365-3156.2012.03040.x 22808917 PMC4099473

[25] Mantel N. The detection of disease clustering and a generalized regression approach. Cancer Res. (1967) 27:209–20. 6018555

[26] Liu T. Epidemiological characteristics and spatial - temporal clustering of COVID-19 in Hebei Province. J Shandong Univ. (2020) 58:74–81. 10.6040/j.issn.1671-7554.0.2020.0745

[27] Zhang Q. Spatial-temporal clustering of cases in a COVID-19 outbreak in Guangzhou city. Chin J Public Health. (2022) 38: 980–4. 10.11847/zgggws1137165

[28] Höhle M. Surveillance: an R package for the monitoring of infectious diseases. Comp Stat. (2007) 22:571–82. 10.1007/s00180-007-0074-8

[29] Li W Du Z Wang Y. Epidemiological characteristics of local outbreak of COVID-19 caused by SARS-CoV-2 delta variant in Liwan district, Guangzhou. Chin J Epidemiol. (2021) 42:1763–8. 10.3760/cma.j.cn112338-20210613-00472 34814609

[30] Campbell F Archer B Laurenson-Schafer H Jinnai Y Konings F Batra N. Increased transmissibility and global spread of SARS-CoV-2 variants of concern as at June 2021. Euro Surveill. (2021) 26:1–6. 10.2807/1560-7917.ES.2021.26.24.2100509 34142653 PMC8212592

[31] Li S Shang L. Epidemiological study of COVID-19 in Shanxi Province. Chin J Nosocomiol. (2020) 30:1152–6. 10.11816/cn.ni.2020-200269

[32] Guan WJ Ni ZY Hu Y Liang WH Ou CQ He JX. Clinical characteristics of coronavirus disease 2019 in China. N Engl J Med. (2020) 382:1708–20. 10.1056/NEJMoa2002032 32109013 PMC7092819

[33] He X Lau EHY Wu P Deng X Wang J Hao X. Temporal dynamics in viral shedding and transmissibility of COVID-19. Nat Med. (2020) 26:672–5. 10.1038/s41591-020-0869-5 32296168

[34] Bai Y Yao L Wei T Tian F Jin DY Chen L. Presumed asymptomatic carrier transmission of COVID-19. JAMA. (2020) 323:1406–7. 10.1001/jama.2020.2565 32083643 PMC7042844

[35] Rothe C Schunk M Sothmann P Bretzel G Froeschl G Wallrauch C. Transmission of 2019-nCoV infection from an asymptomatic contact in Germany. N Engl J Med. (2020) 382:970–1. 10.1056/NEJMc2001468 32003551 PMC7120970

[36] Torjesen I. Covid-19: Delta variant is now UK's most dominant strain and spreading through schools. BMJ. (2021) 373:n1445. 10.1136/bmj.n1445 34088699

[37] Pietrobon AJ Teixeira FME Sato MN I. mmunosenescence and inflammaging: risk factors of severe COVID-19 in older people. Front Immunol. (2020) 11:579220. 10.3389/fimmu.2020.579220 33193377 PMC7656138

[38] He G Rong Z. Comparison of two epidemic patterns of COVID-19 and evaluation of prevention and control effectiveness: an analysis based on Guangzhou andWenzhou. Chin J Epidemiol. (2020) 41:1214–9. 10.3760/cma.j.cn112338-20200303-00242 32244261

[39] Zhang M Xiao JP. transmission dynamics of an outbreak of the COVID-19 delta variant B.1.617.2 — Guangdong Province, China, May–June 2021. China CDC Wkly. (2022) 3:584–6. 10.46234/ccdcw2021.148 34594941 PMC8392962

