# Supplementary material for: Spatial-temporal clustering of an outbreak of SARS-CoV-2 Delta VOC in Guangzhou, China in 2021
Source: Front Public Health. 2022 Dec 9;10:1050096. doi: 10.3389/fpubh.2022.1050096 (PMC9780675; doi:10.3389/fpubh.2022.1050096)
Supplement: Supplementary file 1 [file Data_Sheet_1.docx]

**Supplementary materials**

**Spatial-temporal clustering of an outbreak of SARS-CoV-2 Delta VOC in Guangzhou, China in 2021**


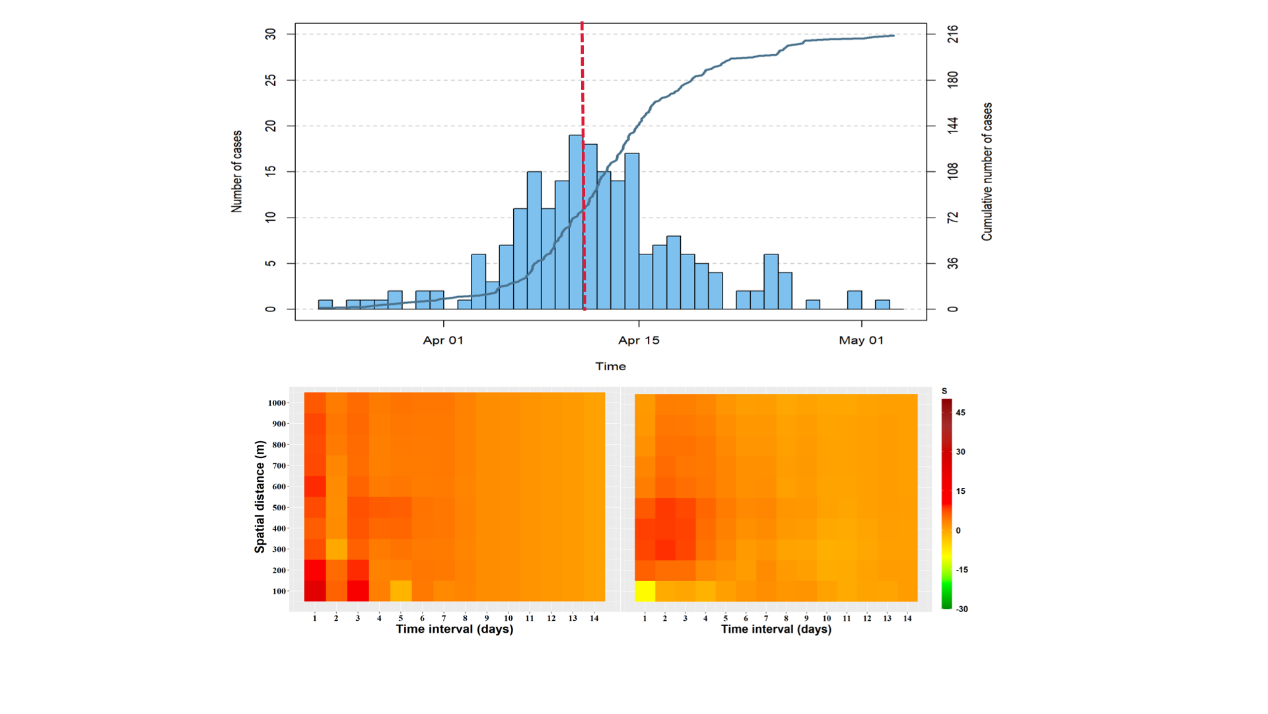


**Figure S1** Spatial-temporal clustering of all cases before and after the peak of the SARS-CoV-2 outbreak in Guangzhou on April 23–May 4, 2020 (1000 m, 1–14 days).

**Table S1** The strength of spatial-temporal clustering (S) of all case pairs before and after the peak of the epidemic in 2021, Guangzhou, China.

| **Time interval (days)** | **Distance between case pairs (meters), 18 May to 31 May 2021** | | | | | | | | | |
| --- | --- | --- | --- | --- | --- | --- | --- | --- | --- | --- |
|  | **100** | **200** | **300** | **400** | **500** | **600** | **700** | **800** | **900** | **1000** |
| 1 | 26.14 | 13.39 | 10.84 | 9.36 | 10.26 | 11.00 | 8.89 | 8.59 | 7.76 | 7.17 |
| 2 | 12.09 | 5.87 | 1.36 | 2.92 | 2.58 | 2.63 | 2.98 | 3.93 | 4.24 | 3.72 |
| 3 | 20.14 | 11.86 | 6.51 | 8.19 | 8.77 | 7.23 | 6.50 | 6.76 | 7.06 | 6.73 |
| 4 | 3.19 | 3.39 | 3.61 | 5.39 | 6.36 | 3.85 | 3.21 | 3.27 | 3.43 | 3.64 |
| 5 | 3.56 | 4.88 | 5.29 | 6.05 | 6.62 | 4.29 | 4.17 | 4.00 | 4.26 | 4.46 |
| 6 | 4.02 | 5.46 | 4.58 | 4.81 | 4.88 | 3.73 | 3.91 | 3.98 | 4.10 | 4.26 |
| 7 | 3.61 | 4.55 | 4.61 | 4.84 | 4.78 | 4.43 | 4.53 | 4.59 | 4.62 | 4.66 |
| 8 | 2.83 | 3.02 | 3.08 | 3.17 | 3.21 | 3.10 | 3.13 | 3.15 | 3.16 | 3.17 |
| 9 | 2.43 | 2.43 | 2.43 | 2.43 | 2.43 | 2.43 | 2.43 | 2.43 | 2.43 | 2.43 |
| 10 | 2.14 | 2.14 | 2.14 | 2.14 | 2.14 | 2.14 | 2.14 | 2.14 | 2.14 | 2.14 |
| 11 | 1.72 | 1.72 | 1.72 | 1.72 | 1.72 | 1.72 | 1.72 | 1.72 | 1.72 | 1.72 |
| 12 | 1.26 | 1.26 | 1.26 | 1.26 | 1.26 | 1.26 | 1.26 | 1.26 | 1.26 | 1.26 |
| 13 | 1.02 | 1.02 | 1.02 | 1.02 | 1.02 | 1.02 | 1.02 | 1.02 | 1.02 | 1.02 |
| 14 | 0.32 | 0.32 | 0.32 | 0.32 | 0.32 | 0.32 | 0.32 | 0.32 | 0.32 | 0.32 |
| **Time interval (days)** | **Distance between case pairs (meters), 1 June to 18 June 2021** | | | | | | | | | |
|  | **100** | **200** | **300** | **400** | **500** | **600** | **700** | **800** | **900** | **1000** |
| 1 | 26.14 | 13.39 | 10.84 | 9.36 | 10.26 | 11.00 | 8.89 | 8.59 | 7.76 | 7.17 |
| 2 | 12.09 | 5.87 | 1.36 | 2.92 | 2.58 | 2.63 | 2.98 | 3.93 | 4.24 | 3.72 |
| 3 | 20.14 | 11.86 | 6.51 | 8.19 | 8.77 | 7.23 | 6.50 | 6.76 | 7.06 | 6.73 |
| 4 | 3.19 | 3.39 | 3.61 | 5.39 | 6.36 | 3.85 | 3.21 | 3.27 | 3.43 | 3.64 |
| 5 | 3.56 | 4.88 | 5.29 | 6.05 | 6.62 | 4.29 | 4.17 | 4.00 | 4.26 | 4.46 |
| 6 | 4.02 | 5.46 | 4.58 | 4.81 | 4.88 | 3.73 | 3.91 | 3.98 | 4.10 | 4.26 |
| 7 | 3.61 | 4.55 | 4.61 | 4.84 | 4.78 | 4.43 | 4.53 | 4.59 | 4.62 | 4.66 |
| 8 | 2.83 | 3.02 | 3.08 | 3.17 | 3.21 | 3.10 | 3.13 | 3.15 | 3.16 | 3.17 |
| 9 | 2.43 | 2.43 | 2.43 | 2.43 | 2.43 | 2.43 | 2.43 | 2.43 | 2.43 | 2.43 |
| 10 | 2.14 | 2.14 | 2.14 | 2.14 | 2.14 | 2.14 | 2.14 | 2.14 | 2.14 | 2.14 |
| 11 | 1.72 | 1.72 | 1.72 | 1.72 | 1.72 | 1.72 | 1.72 | 1.72 | 1.72 | 1.72 |
| 12 | 1.26 | 1.26 | 1.26 | 1.26 | 1.26 | 1.26 | 1.26 | 1.26 | 1.26 | 1.26 |
| 13 | 1.02 | 1.02 | 1.02 | 1.02 | 1.02 | 1.02 | 1.02 | 1.02 | 1.02 | 1.02 |
| 14 | 0.32 | 0.32 | 0.32 | 0.32 | 0.32 | 0.32 | 0.32 | 0.32 | 0.32 | 0.32 |

**Table S2** Changes in strength (S) of spatial-temporal clustering before and after the peak of outbreaks in Guangzhou in 2020 and 2021

| Outbreak | Temporal variable (days) | S-pre | S-post | S-pre | S-post | S-change | S-change rate |
| --- | --- | --- | --- | --- | --- | --- | --- |
|  |  | (Accumulative) | (Accumulative) | (Median) | (Median) |  |  |
| 2021 | 1–5 | 332.4 | 203.21 | 5.63 | 4.32 | 129.19 | 38.87% |
|  | 6–10 | 165.65 | 79.04 | 3.14 | 1.45 | 86.61 | 52.28% |
|  | 11–14 | 43.22 | 22.35 | 1.14 | 0.66 | 20.87 | 48.29% |
| 2020 | 1–5 | 279.21 | 195.4 | 5.19 | 4.1 | 83.81 | 30.02% |
|  | 6–10 | 158.86 | 51.09 | 3.2 | 0.99 | 107.77 | 67.84% |
|  | 11–14 | 44.04 | 14.7 | 1.14 | 0.54 | 29.34 | 66.62% |

*S-change rate=100%*(S-pre – S-post)/ S-pre
